# Associations Among Food Delay of Gratification, Cognitive Measures, and Environment in a Community Preschool Sample

**DOI:** 10.3389/fnut.2022.788583

**Published:** 2022-04-11

**Authors:** Nicole R. Giuliani, Nichole R. Kelly

**Affiliations:** ^1^Department of Special Education and Clinical Sciences, Prevention Science Institute, University of Oregon, Eugene, OR, United States; ^2^Department of Counseling Psychology and Human Services, Prevention Science Institute, University of Oregon, Eugene, OR, United States

**Keywords:** delay of gratification, cognitive measures, executive function, preschool, environment

## Abstract

Much of the work on the development of appetite self-regulation in early childhood employs tasks assessing Delay of Gratification (DoG). While this skill is thought to rely on “cool” cognitive processes like effortful control, executive functioning, and self-regulation, demonstration of how laboratory measures of food DoG relate to common assessments of those cognitive processes in community samples of children is needed. This study presents secondary data investigating the associations between two laboratory tasks of food DoG, the Snack Delay and Tongue Tasks, and an array of laboratory and parent-report cognitive measures in a sample of 88 children ages 3-6 (*M* age = 4.05, SD = 0.76), as well as how four measures of the child's environment were associated with food DoG. Results indicated that both measures of food DoG were positively correlated with performance on the cognitive tasks, with stronger associations observed for the Tongue Task. Family income was positively associated with food DoG as measured by the Tongue Task, and child negative life events in the past year were negatively correlated with food DoG as measured by the Snack Delay Task. These findings present the pattern of associations between cognitive tasks and food DoG, the development of which may be meaningfully affected by specific aspects of family environment.

## Introduction

Delay of gratification (DoG) refers to an individual's ability to forego an immediate reward in favor of a later, larger reward. While DoG can be applied to various rewards, many behavioral paradigms use food stimuli to measure this construct in preschool-aged children (1–3). This is referred to in the literature as food-related, or appetite, self-regulation (2). While some of the main cognitive mechanisms that enable successful food DoG in early childhood have been identified in previous studies [e.g., effortful control, executive function; see (1, 4)], the measures used to assess these mechanisms vary. Indeed, a wide array of assessment tools are used in the literature to measure these constructs in early childhood; it remains unclear the degree to which these measures capture those constructs and how they relate to DoG performance. To address this gap in the literature, the present study employed data collected as part of a larger study to investigate the associations between multiple measures of food DoG and tasks assessing theoretically relevant cognitive constructs.

### Delay of Gratification

In the decades of work that have been done on DoG, researchers have separately conceptualized it as measuring (a) sensitivity to reward value, (b) impulsivity, and (c) top-down regulatory control (5). While many models of self-regulation situate top-down, cognitive processes in dynamic interaction with bottom-up reactions to stimuli [e.g., (6–9)], it may be that DoG itself represents the entire process. Specifically, DoG behavior captures the degree to which top-down, cognitive processes are engaged with the goal of regulating bottom-up reactions to a reward, such that delay behavior results from the balance achieved between the two systems. Indeed, DoG depends on “the cognitive and attentional mechanisms that help execute goal directed behavior” (7). Many such cognitive mechanisms have been evoked with regard to successful DoG, including effortful control [EC; (10, 11)] and executive function [EF; (12–14)]. Interestingly, while EF and EC stem from different traditions, they are thought to represent overlapping processes (15) and the same tasks are used to assess them [e.g., Day/Night, Go/NoGo Tasks; (16, 17)].

However, other conceptualizations of EF may get closer to capturing the type of cognitive processes engaged during DoG. Much of the recent work on DoG treats it as a form of “hot” EF (18), which is “involved in social and affective situations that generate emotion and motivation, as well as tension between immediate gratification and greater long-term reward” (19). Use of DoG in food contexts can be particularly evocative, as food can be rewarding, induce impulsive behavior, and be emotional for many people (2, 20). There is some evidence that, compared to non-food rewards, food DoG is uniquely associated with weight in early childhood (21), supporting the investigation of food DoG in this age range. Indeed, hot and cool forms of EF are thought to follow distinct but related trajectories in middle childhood (19), but it remains unclear the degree to which successful food DoG is associated with measures of cool EF earlier in childhood.

### Delay of Gratification in Context

Ecological systems models stress the importance of interactions between biological and environmental factors in explaining development (22). To this end, a large body of literature demonstrates the effect that the family environment has on EC and EF development [e.g., (23–26)], as well as on food DoG [e.g., (3, 27)]. Indeed, this literature suggests that the resources and stressors in the child's environment have a meaningful effect on DoG development. However, there are relatively few places in the literature presenting simple associations between different aspects of family environment and multiple measures of food DoG in preschool-aged children. The present data set provides us with the opportunity to address this gap in the literature.

In the literature on environmental influences on the development of food DoG, several candidate measures emerge. First, socioeconomic status (SES) is positively associated with better performance on DoG tasks [e.g., (3, 28)] and other measures of food-related self-regulation [e.g., (29)]. As such, both family income and maternal education—common measures of SES—should positively correlate with delay time. Second, environmental stressors beyond low SES are also associated with the development of EF and DoG (1, 30, 31). Two measures of environmental stress in the present dataset, maternal depression and negative life events experienced by the child, are risk factors for high weight in children (32–35) with food-related self-regulation proposed as a mechanism (36). Extant research suggests that maternal depression is negatively associated with food DoG in children [e.g., (37)]. Similarly, experiencing stressful life events such as losing one's housing to an earthquake has been associated with decreased DoG (38). This is consistent with a “fast life history strategy,” where environmental uncertainty promotes seeking immediate gratification (39).

### The Present Study

The present study uses data collected as part of the Parent-Child Self-Regulation study (40). While the main focus of the original study was to quantify associations between parent and child measures of food-related DoG and attentional and inhibitory control, we also gathered additional measures that have not yet been published.

Here, we present secondary analyses addressing the aforementioned gaps in the literature regarding the associations among (1) food DoG and cognitive measures, and (2) food DoG and measures of family income, maternal education, maternal depression, and recent child negative life events in a community sample of typically-developing 3-6 year old children.

## Methods

### Participants

Families were recruited via online flyers; criteria for participation were biological mothers over age 18 with children ages 3 through 5 who had not yet entered kindergarten at the time of assessment. Non-inclusion criteria were if mothers had less than half-time custody of the child, had a history of significant neurological disorder(s), or were taking medication that affects cognitive function; if the child had a developmental delay, sensory impairment, or the mother believed the child could not participate in the study successfully; or if the family was involved with child welfare services or reported that their primary language was not English. All study procedures were approved by the University's Committee for the Protection of Human Subjects.

This study presents data from 88 children ages 3–6 (*M* age = 4.05, SD = 0.76; Table 1). These data are from a larger study designed to investigate self-regulation in parents and children, parent-child interactions, parent feeding practices, and child eating behavior. Data from this sample have been described in Giuliani and Kelly (41) and Giuliani et al. (40).

**Table 1:** Demographic information.

**Demographics**	***M* (SD)**	**%**
**Child demographics**		
Age (years)	4.05 (0.76)	
Female Race or Ethnicity		49%
White		87.23%
Asian		2.13%
Hispanic		0%
Multiracial		8.51%
Native American/Indian		2.13%
Preschool attendance		61.7%

### Protocol

Mothers and children came into the laboratory for a roughly 3-h visit consisting of video-recorded parent-child interactions, mother-completed surveys, and child assessments. Measures relevant to the present analyses are described below. Families were paid $60 for their time.

### Measures

#### Food Delay of Gratification Tasks

##### Snack Delay Task

In this task (40, 41), children were asked to choose a preferred snack (choices: fruit snacks, M&Ms, goldfish crackers). The experimenter placed the snack on a napkin in front of the child and asked them to wait until the experimenter rang a bell before retrieving it. The child was then told that they would receive a second snack if they were able to wait until the bell was rung. Four trials were conducted, where the child had to wait 30, 60, 120, and 180 s for the bell to ring. Halfway through each trial, the experimenter picked up the bell as if they were about to ring it. For each trial, the child was given a score representing waiting behavior: 0 (eats snack before bell is lifted), 1 (eats snack after bell is lifted), 2 (touches bell/snack before bell is lifted), 3 (touches bell/snack after bell is lifted), or 4 (waits for bell to ring before touching snack/bell). The final score was the average score over four trials, such that a child with an average score of 0 ate the snack before the bell was lifted for all trials, and a child with an average score of 4 waited until the bell was rung for all trials. This task has a 1–2 week test-retest reliability of 0.5 (42).

##### Tongue Task

As in the Snack Delay Task, the Tongue Task started with the child choosing a preferred snack. The child was then asked to place the snack on their tongue, and were told to wait until a bell was rung to eat it. Four trials were administered (10, 20, 30, 15 s), and coded to reflect the length of time before the child ate the snack. The final score was the average score across the four trials. Preschool-aged Fall-Spring academic year test-retest reliability as part of a larger hot EF composite was estimated at 0.58 (43).

#### Cognitive Tasks

##### Flanker Task

The Flanker Task was administered via the NIH Toolbox (44). Children were presented with a stimulus on the center of a tablet screen and were required to indicate the left-right orientation while inhibiting attention to the stimuli flanking it. On some trials the orientation of the flankers was congruent with the orientation of the central stimulus and on the other trials the flankers were incongruent. The test consisted of a block of 20 fish trials and a block of 20 arrow trials, shown only if the participant scored >90% on the fish stimuli. The NIH Toolbox uses a two-vector method to compute performance, which incorporated both accuracy and reaction time for participants who maintained a high level of accuracy (>80% correct), and accuracy only for those who did not meet this criterion. This computed score was used to represent performance (40). This task has a 7–21 day test-retest reliability of 0.89 (44).

##### Go/NoGo Tasks

Two GNG tasks were administered to children. First, children performed the Zoo Game (45). The task asks children to help a zookeeper put animals back in their cages by pressing a button as quickly as they can [Go (G) trials], unless they see the monkey helping the zookeeper [NoGo (NG) trials]. It begins with three practice blocks in which children can practice (1) pressing the laptop button when they see an animal, (2) pressing the button within a certain time limit, and (3) inhibiting their response when they see the monkey. Feedback at the end of each trial presented children with a smiling face if they correctly withheld their response on NG trials and a mad face if they either pressed the button on NG trials or did not press the button on G trials. Each trial consisted of a 500–700 ms jittered fixation cross, 1200 ms stimulus presentation, 500 ms black screen, and 1,000 ms feedback. Responses could be made while the stimulus was on screen or at any point during the following 500 ms. A total of 90 trials were completed, 25% of which were NG. Percent correct was calculated across both types of trials. Two-to-four week test-retest reliability of a similar task was 0.58 (46).

We also asked children to complete the Fish GNG Task from the Early Years Toolbox (47). The task asks children to respond to G trials (“catch fish,” 80%) and withhold responding on NG trials (“avoid sharks,” 20%). The task begins with go instructions followed by 5 practice G trials, NoGo instructions followed by 5 practice NG trials, combined GNG instructions followed by a mixed block of 10 practice trials (80% G), and a recap of instructions prior to the task commencing. Auditory feedback was provided on all practice trials. The task itself did not contain feedback, and was comprised of 75 stimuli over three blocks. Stimuli were presented in pseudo-random order, such that a block never began with a NG stimulus and no more than two successive trials were NG stimuli, separated by a 1,000 ms inter-stimulus-interval. Percent correct was calculated across both types of trials. Due to computer error, data from 15 participants were not recorded. The split-half reliability of this task was 0.84 in the original validation sample (47).

We originally planned on combining across the two GNG tasks in previous analyses using these data (40). However, the relatively modest correlation between the two tasks (*r* = 0.44, *p* < 0.001) suggests that they may index related but separate processes. Therefore, we opted to consider the two tasks separately.

##### Day/Night Stroop Task

In this task (16), the child was shown a total of 16 pictures in a random sequence that depict either a moon on a dark background or a sun on a white background. When the child was shown the picture of the sun or moon, they were instructed to say the opposite time of day. For instance, if the child was shown a picture of the sun, they should have said “night.” The total number of correct responses was recorded, and percent correct was calculated. This task has a 2-week test-retest reliability of 0.84 (48).

##### Balance Beam Task

In this task (49), which is sometimes called “Walk-a-Line-Slowly,” a 12 ft piece of tape was placed on the floor. The child was instructed to walk along the tape, once at regular speed, and twice slowly. This experimenter recorded and coded the times for each trial in seconds. Difference scores between the average of the two slow times and the regular time was calculated. This task has a Fall-Spring academic year test-retest reliability of 0.42 (43).

##### Tower Task

In this task (50), the child was asked to take turns with the experimenter in building a tower. Twenty wooden blocks were used, with 10 blocks allocated to each person. The experimenter deliberately waited to place their block until the child explicitly signaled that they were giving a turn. The child earned 1 point for each time they appropriately gave a turn to the experimenter. If the child gave the experimenter all their due turns, the child earned up to 10 points. The child could also gain one point for arranging the tower to prevent it from collapsing, and for waiting 10 s after placing their block even if they did not explicitly signal that they were giving a turn to the experimenter. Points were summed to create a final score for this task. This task has a 1–2 week test-retest reliability of 0.85 (42).

##### Head-Toes-Knees-Shoulder Task

In this task (51), children were provided with paired behavioral rules (e.g., touch your head/touch your toes) and then asked to do the opposite. First, the child completed 10 trials where they were asked to touch their head or their toes. If the child responded correctly to 5 or more items, then the second set of paired rules (touch your shoulders/touch your knees) was introduced. If the child produced the correct response immediately, the item was scored 2. If the child self-corrected without prompting, the item was scored 1. If they did not touch the correct part of their body, the item was scored 0; all points summed to create a final score. This task has a Fall-Spring academic year test-retest reliability of 0.6 in a pre-kindergarten sample (52).

#### Family Demographics

Mothers were asked to report the birth date, sex, race, and ethnicity of their child. From that, age was calculated as the number of days between the child's birth and the session date, divided by 365.25. Mothers also reported the gross family income in US$ and her highest level of educational attainment by degree. Degree earned was then transformed into years of education, where high school diploma or GED = 12, Associate = 14, Bachelor's = 16, Master's = 18, and Doctoral = 22.

#### Mother-Report Surveys

Mothers completed the Devereux Early Childhood Assessment for Preschoolers–Second Edition [DECA; (55)], from which we used the Self-Regulation (SR) subscale (α = 0.87). We also administered the Child Behavior Questionnaire–Very Short Form [CBQ-VSF; (56)], from which we used the Effortful Control (EC) subscale (α = 0.64).

Mothers also completed the Center for Epidemiological Studies Depression [CESD; (57)] scale (α = 0.91) and a modified version of the Coddington Life Events Questionnaire (58) to report their depressive symptoms and their child's negative life events in the past year, respectively.

### Analyses

For all variables, outliers were Winsorized (59) at 3 standard deviations from the mean (noted in Table 2) and then assessed for skew and kurtosis. Gross family income; maternal depressive symptoms; performance on the Snack Delay, Tongue, Zoo Go/NoGo, Flanker, Day/Night Stroop, Balance Beam, Tower, and HTKS Tasks; and child negative life events in the past year were identified as non-normally distributed (skewness and/or kurtosis > ±1). To maximize sample size and statistical variance, we opted to retain Winsorized values and use non-parametric statistical tests that did not assume normality. Analyses of both the raw data and the data with outlier cases removed did not meaningfully change the results, indicating that extreme but plausible values did not drive the study's findings.

**Table 2:** Descriptive data of self-regulation and family environment variables.

**Variable**	***N***	***M***	***SD***	**Observed Range**
Snack delay task	88	2.01	1.66	0–4.00
Tongue task	85	15.65	5.66	0.63–18.75
Flanker task	81	2.52	1.91	0–7.06
Fish Go/NoGo task^*^	66	0.66	0.17	0.01–1.00
Zoo Go/NoGo task^*^	83	51.68	14.39	8.22–68.24
Day/Night stroop task	83	65.29	34.72	0–100.00
Balance beam task^*^	88	3.04	4.91	−5–21.57
Tower task	86	6.57	3.60	0–10.00
HTKS task	82	19.43	18.67	0–52.00
CBQ-VSF EC subscale	87	5.36	0.64	4–6.58
DECA SR subscale^*^	87	33.56	4.57	18–45.00
Gross family income (US$)	86	69,329.00	48,754.00	0–260,000.00
Maternal years of education	88	15.15	2.47	8–22.00
Maternal depression symptoms (CES-D)	88	9.67	8.80	0–38.00
Child negative events–past year (CLEQ)	87	2.31	2.24	0–10.00

*HTKS, Head-Toes-Knees-Shoulders Task; CBQ-VSF EC, Child Behavior Questionnaire (Very Short Form) Effortful Control subscale; DECA SR, Devereux Early Childhood Assessment Self-Regulation subscale; CES-D, Center for Epidemiological Studies–Depression scale; CLEQ, Coddington Life Events Questionnaire*.

* *indicates variable Winsorized at 3 standard deviations from the mean for analyses; uncorrected values are presented here*.

All analyses were run using R (60). For both aims, associations were measured using Spearman's correlations. All analyses were adjusted for multiple tests by hypothesis, using the Benjamini-Hochberg correction (53); adjusted *p*-values are presented. Correlations were also disattenuated to account for varying measure reliability using the reliability estimates provided in the measures descriptions above (61). Formal comparisons of the strength of the correlations values were evaluated using https://www.psychometrica.de/ (62).

## Results

Descriptive statistics for task variables and measures of family environment are presented in Table 2.

### Zero-Order Associations

After adjusting for multiple comparisons, zero-order correlations (Table 3) revealed that performance on the Snack Delay and Tongue DoG Tasks was significantly positively correlated, *r*_(85)_ = 0.43, *p* < 0.001, 95% CI [0.24, 0.59]. Both DoG tasks were significantly positively correlated with performance on the Flanker, Fish GNG, Day/Night Stroop, Tower, and HTKS Tasks (*r*-values: 0.25–0.54, *p*-values <0.05, see Table 3 for 95% CIs). For the Zoo GNG and Balance Beam Tasks, only the Tongue Task was significantly correlated (*r*-values: 0.29–0.36, *p*-values <0.05, see Table 3 for 95% CIs). With regard to the mother-report surveys, only the DECA SR subscale and Snack Delay Task were significantly correlated, *r*_(87)_ = 0.26, *p* = 0.03, 95% CI [0.05, 0.44]. Direct comparisons of the associations between each of the cognitive variables and the food DoG tasks revealed that the associations were stronger between the Tongue Task and the Zoo GNG, Balance Beam, HTKS, and CBQ-VSF EC compared to the Snack Delay Task and each of those measures (*p*-values <0.05).

**Table 3:** Correlations among self-regulation variables.

**Variable**	**1**	**2**	**3**	**4**	**5**	**6**	**7**	**8**	**9**	**10**	**11**
1. Snack Delay		0.80 [0.70, 0.86]	0.41 [0.21, 0.58]	0.48 [0.27, 0.65]	0.37 [0.16, 0.54]	0.49 [0.31, 0.64]	0.37 [0.17, 0.54]	0.58 [0.41, 0.70]	0.45 [0.26, 0.61]	0.14 [-0.07, 0.34]	0.39 [0.19, 0.55]
2. Tongue	0.43^**^ [0.24, 0.59]		0.67 [0.53, 0.78]	0.77 [0.65, 0.86]	0.50 [0.32, 0.65]	0.72 [0.60, 0.81]	0.73 [0.61, 0.81]	0.69 [0.56, 0.79]	0.91 [0.86, 0.94]	0.31 [0.10, 0.49]	0.18 [-0.04, 0.38]
3. Flanker	0.27^*^ [0.06, 0.46]	0.48^**^ [0.29, 0.64]		0.66 [0.49, 0.78]	0.84 [0.76, 0.89]	0.56 [0.39, 0.70]	0.92 [0.88, 0.95]	0.52 [0.34, 0.67]	0.83 [0.74, 0.89]	0.12 [-0.10, 0.33]	0.00 [-0.22, 0.22]
4. Fish Go/NoGo	0.31^*^ [0.08, 0.52]	0.54^**^ [0.34, 0.69]	0.57^**^ [0.37, 0.72]		0.75 [0.62, 0.84]	0.60 [0.42, 0.74]	0.69 [0.53, 0.80]	0.53 [0.32, 0.68]	0.67 [0.50, 0.78]	−0.24 [-0.46, 0.00]	0.05 [-0.20, 0.29]
5. Zoo Go/NoGo	0.20 [-0.02, 0.40]	0.29^*^ [0.08, 0.48]	0.60^**^ [0.44, 0.73]	0.52^**^ [0.32, 0.68 ]		0.63 [0.48, 0.75]	0.69 [0.55, 0.78]	0.52 [0.34, 0.66]	0.95 [0.92, 0.97]	0.03 [-0.19, 0.25]	0.09 [-0.13, 0.30]
6. Day/Night	0.32^*^ [0.11, 0.50]	0.51^**^ [0.32, 0.65]	0.48^**^ [0.29, 0.64]	0.51^**^ [0.30, 0.67]	0.44^**^ [0.24, 0.60]		0.95 [0.92, 0.96]	0.53 [0.36, 0.67]	0.79 [0.70, 0.86]	0.05 [-0.17, 0.27]	0.16 [-0.06, 0.36]
7. Balance Beam	0.17 [-0.04, 0.37]	0.36^**^ [0.16, 0.53]	0.56^**^ [0.39, 0.70]	0.41^**^ [0.18, 0.59]	0.34^**^ [0.13, 0.52]	0.56^**^ [0.39, 0.69]		0.69 [0.56, 0.79]	0.85 [0.77, 0.90]	−0.05 [-0.26, 0.16]	0.11 [-0.11, 0.31]
8. Tower	0.37^**^ [0.18, 0.54]	0.48^**^ [0.30, 0.63]	0.46^**^ [0.26, 0.61]	0.44^**^ [0.22, 0.62]	0.37^**^ [0.16, 0.54]	0.45^**^ [0.26, 0.61]	0.41^**^ [0.22, 0.57]		0.76 [0.64, 0.84]	0.05 [-0.16, 0.26]	0.24 [0.03, 0.43]
9. HTKS	0.25^*^ [0.03, 0.44]	0.53^**^ [0.35, 0.67]	0.60^**^ [0.44, 0.73]	0.47^**^ [0.26, 0.64]	0.56^**^ [0.38, 0.69]	0.56^**^ [0.39, 0.69]	0.43^**^ [0.23, 0.59]	0.54^**^ [0.37, 0.68]		0.05 [-0.17, 0.27]	-0.03 [-0.25, 0.19]
10. CBQ-VSF EC	0.08 [-0.13, 0.29]	0.19 [-0.03, 0.39]	0.09 [-0.12, 0.31]	−0.18 [-0.40, 0.07]	0.02 [-0.20, 0.24]	0.04 [-0.18, 0.25]	-0.03 [-0.24, 0.18]	0.04 [-0.18, 0.25]	0.03 [-0.19, 0.25]		0.51 [0.33, 0.65]
11. DECA SR	0.26^*^ [0.05, 0.44]	0.13 [-0.09, 0.33]	0.00 [-0.22, 0.22]	0.04 [-0.2, 0.28]	0.06 [-0.16, 0.28]	0.14 [-0.08, 0.34]	0.06 [-0.15, 0.27]	0.21 [-0.01, 0.40]	-0.02 [-0.24, 0.20]	0.38^**^ [ 0.18, 0.54]	

*Statistics below the diagonal are Spearman correlations with 95% confidence intervals shown in brackets. Significance tests are corrected for multiple comparisons using the Benjamini-Hochberg (53) method. Statistics above the diagonal show disattenuated correlations with 95% confidence intervals in brackets. Correlations were disattenuated using the following reliability estimates: Snack Delay, 0.55 (42); Tongue Task, 0.58 (54); Flanker Task, 0.89 (44); Fish Go/NoGo, 0.84 (47); Zoo Go/NoGo, 0.58 (46); Day/Night Task, 0.84 (48); Balance Beam Task, 0.42 (43); Tower Task, 0.85 (42); HTKS Task, 0.6 (52); CBQ-VSF EC, 0.64 (present sample); and DECA SR, 0.87 (present sample). HTKS, Head-Toes-Knees-Shoulders Task. CBQ-VSF EC, Child Behavior Questionnaire (Very Short Form) Effortful Control subscale. DECA SR, Devereaux Early Childhood Assessment Self-Regulation subscale*.

** *p < 0.001*.

* *p < 0.05*.

After disattenuating the correlations to account for measure reliability, the correlation between the Snack Delay and Tongue Tasks increased from 0.43 to 0.80, 95% CI [0.70, 0.86]. All correlations between laboratory measures were significant at *p* < 0.05. The pattern of significant correlations between the laboratory and mother-report surveys remained the same. Lastly, direct comparisons of the associations between each cognitive variable and the two food DoG tasks showed that the correlations between the cognitive measures and the Tongue Task were all significantly stronger than those between the cognitive measures and the Snack Delay Task.

### Associations Between DoG Tasks and Family Environment

After adjusting for multiple comparisons, family income was significantly positively associated with performance on the Tongue Task, *r*_(83)_ = 0.40, *p* = 0.001, 95% CI [0.20, 0.56] (Table 4). Children from families with higher yearly gross incomes performed better on the Tongue Task. The positive association between family income and Snack Delay Task performance was not statistically significant (*p* = 0.0501). Maternal years of education was not significantly associated with performance on either food DoG task (Snack Delay Task: *p* = 0.27; Tongue Task: *p* = 0.0501). The associations between SES measures and Tongue Task performance were significantly stronger than those between SES measures and Snack Delay Task performance (*p*-values <0.05).

**Table 4:** Correlations between measures of delay of gratification and family environment.

**Variable**	**1**	**2**	**3**	**4**	**5**
1. Snack delay task					
2. Tongue task	0.43^**^ [0.24, 0.59]				
3. Family income ($)	0.23 [0.02, 0.42]	0.40^**^ [0.20, 0.56]			
4. Maternal education (years)	0.12 [-0.09, 0.33]	0.24 [0.03, 0.43]	0.58^**^ [0.42, 0.70]		
5. Maternal depression (CES-D total)	-0.15 [-0.35, 0.05]	−0.19 [-0.39, 0.02]	-0.29^*^ [-0.47,−0.08]	−0.16 [-0.36, 0.06]	
6. Child negative life events in past year (CLEQ)	-0.29^*^ [-0.48,−0.09]	0.00 [-0.21, 0.21]	-0.28^*^ [-0.47,−0.08]	−0.23 [-0.42,−0.02]	0.28^*^ [0.07, 0.47]

*Statistics are Spearman correlations with 95% confidence intervals shown in brackets. CES-D, Center for Epidemiological Studies–Depression; CLEQ, Coddington Life Events Questionnaire. All p-values corrected for multiple comparisons using the procedure of Benjamini-Hochberg (53)*.

** *p < 0.001*.

* *p < 0.05*.

Child negative life events in the past year was significantly negatively associated with performance on the Snack Delay Task, *r*_(87)_ = −0.29, *p* = 0.019, 95% CI [-0.47,−0.09], such that children who experienced more recent negative life events did not wait as long for the second snack as compared to children who had experienced fewer negative life events. There was not a significant association between child negative life events and Tongue Task performance (*p* > 0.05), nor were there significant associations between mother-reported depressive symptoms and performance on either task (*p*-values > 0.05). The association between child negative life events and Snack Delay performance were significantly stronger than the association with Tongue Task performance (*p*-values <0.001).

## Discussion

The purpose of the present study was to present associations among two commonly-used measures of food DoG and an array of cognitive measures in a community sample of preschool-aged children, and explore the degree to which food DoG was associated with four measures family environment thought to play a role in DoG development.

### Food DoG and Cognitive Measures

Performance on both food DoG tasks was significantly positively associated with performance on the cognitive tasks in this data set. Like most tasks used to assess EC and EF, tasks used in the current study suffer from task impurity, in that successful performance is dependent on multiple cognitive processes (63, 64). However, while EF is broadly implicated in eating behavior in young children [e.g., (9)], previous analyses on the present sample directly compared the degree to which food DoG (Snack Delay), attentional control (Flanker) and inhibitory control (GNG) predicted later EAH. Here, we found that only food DoG significantly predicted later EAH (41), indicating that this hot EF measure may better capture the food-related regulatory processes recruited when making food choices in the absence of hunger.

Compared to the laboratory assessments, the two mother-report measures showed a different pattern. Even after disattenuating the correlations to account for measure reliability, we found that the CBQ-VSF EC subscale positively correlated with performance on the Tongue Task only, whereas the DECA SR subscale was positively correlated with the Snack Delay Task and the Tower Task. The two mother-report measures were positively correlated with each other, a pattern that suggests some common method variance. This may be due to known low levels of convergence between survey and behavioral measures of EF, which could indicate that the types of assessments reflect different underlying mechanisms, or could be simply due to the differing method of measurement [e.g., (29, 65)]. Regardless, the finding that mother-reported EC was positively correlated with Tongue Task performance and mother-reported SR [which includes EF; see (66)], suggests that the two food DoG tasks may vary slightly in their underlying cognitive bases—with the Tongue Task relying more on EC and the Snack Delay relying more on EF. However, this remains to be tested empirically.

### Food DoG and Family Environment

Our investigations into how the food DoG tasks were associated with measures of family environment were mostly consistent with the extant literature. First, the overall qualitative pattern showed positive associations between measures of family SES (i.e., income, maternal education) and food DoG. This is in line with research showing that individuals who have more resource certainty perform better on DoG tasks (28, 67). Of the four correlations, the only one that rose to the level of significance was the association between family income and performance on the Tongue Task. This may be due to the increased temptation of holding a desired treat on one's tongue in the Tongue Task, as opposed to simply looking at it as is done in the Snack Delay.

Second, with regard to measures of environmental stress, negative associations between maternal depression and food DoG were not significant. While in the same direction as the empirical and theoretical literature stating that maternal depression predicts poorer child food DoG [e.g., (37, 68)], the non-significant association seen in the present data may be due to the fact that we used a low-risk, community sample. Specifically, the CES-D ranges from 0 to 60, with a clinical cutoff of 16 (69). Our sample ranged from 0 to 38, with a mean of 9.67. Indeed, only 19 of the 87 mothers scored 16 or above on the CES-D. We did see, however, a significant negative correlation between recent child negative life events and performance on the Snack Delay Task, such that more negative life events were associated with shorter delay time. This is consistent with Life History Theory, where a lower sense of control is associated with a decreased willingness to delay gratification (67). While a sense of control can vary by person and situation, it may be that a large number of recent negative life events imparts a general sense of uncontrollability for a young child, thus motivating them to choose the sooner, more certain reward.

### Limitations, Conclusions, and Future Directions

In addition to the ones listed above, this study had several limitations. First, this data set did not include measures of working memory or non-food DoG, which would help us better understand the extent to which these results capture EF and DoG more generally. Second, recent work has shown that the use of reaction time differences as measures of Flanker Task performance can be unreliable (70), and as such these results should be interpreted with caution. Third, this was a relatively racially-homogeneous, low-risk, community sample of families; as such, these data may not be generalizable to other samples. While we did have reasonable variance in our measures of family environment, children raised in higher-risk environments may show different associations between those measures and food DoG. Fourth, we observed differences in the pattern of significant findings for the two GNG Tasks, which may be because the Zoo version employs a greater variety of stimuli than the Fish version and thus requires more working memory (71). Lastly, we did not include any measures of observed parenting behavior in these analyses, which would be useful with regard to better understanding how food DoG relates to environmental context.

These findings are meaningful to the literature in two ways. First, the patterns of associations between food DoG and the cognitive measures in this study inform the ongoing discussion on how to situate DoG in the family of related constructs. Our results suggest that, compared to the more popular Snack Delay Task, the Tongue Task may be a better way to measure hot EF in the context of food DoG, as it is more consistently correlated with performance on non-food cognitive tasks. However, future work using tasks that more clearly recruit separate cognitive processes [e.g., working memory, cognitive flexibility, behavioral inhibition; (64)] is needed to determine the degree to which different food DoG tasks rely on separate underlying cognitive constructs. Second, the present findings support and add to the literature on environmental influences on DoG development. Specifically, we found that family income and child negative life events are meaningfully associated with food DoG, in directions that are consistent with the literature. These results stress the role that childhood resource certainty and controllability may have on the development of DoG. Taken together, these results demonstrate the degree to which an array of common cognitive measures are associated with food DoG, the development of which may be meaningfully affected by specific aspects of family environment.

## Data Availability Statement

The datasets presented in this study can be found in online repositories. The names of the repository/repositories and accession number(s) can be found below: https://osf.io/dfmhe/.

## Ethics Statement

The studies involving human participants were reviewed and approved by Institutional Review Board of the University of Oregon. Written informed consent to participate in this study was provided by the participants' legal guardian/next of kin.

## Author Contributions

NG and NK designed the study, edited drafts, and approved the final version. NG collected and analyzed the data and wrote the manuscript. Both authors contributed to the article and approved the submitted version.

## Funding

This study was funded by an Early Career Research Award from the Society for the Study of School Psychology to NG.

## Conflict of Interest

The authors declare that the research was conducted in the absence of any commercial or financial relationships that could be construed as a potential conflict of interest.

## Publisher's Note

All claims expressed in this article are solely those of the authors and do not necessarily represent those of their affiliated organizations, or those of the publisher, the editors and the reviewers. Any product that may be evaluated in this article, or claim that may be made by its manufacturer, is not guaranteed or endorsed by the publisher.
